# Emotion-focused therapy and forgiveness in the older population: Protocol for a feasibility randomized controlled trial

**DOI:** 10.1371/journal.pone.0345792

**Published:** 2026-05-13

**Authors:** Bernardo Corrêa d’Almeida, Carla Cunha

**Affiliations:** 1 Department of Social and Behavioural Sciences, University of Maia‌‌, Maia‌‌, Portugal; 2 Center for Psychology at University of Porto (CPUP)‌‌, Porto, Portugal; King Khalid University, EGYPT

## Abstract

Over recent decades, Emotion-Focused Therapy, aging, and forgiveness have garnered significant attention in the field of psychology. However, there is a lack of studies on Emotion-Focused Therapy and forgiveness specifically tailored for older adults. This article presents a protocol designed to assess the feasibility and acceptability of a randomized controlled trial of Emotion-Focused Therapy aimed at resolving emotional injuries in individuals aged 65 and older, within the context of interpersonal offenses. Feasibility will be evaluated through recruitment and retention rates, as well as adherence to the intervention protocol. Acceptability will be assessed based on perceived treatment credibility, participants’ expectations regarding outcomes, levels of engagement, and overall satisfaction with the intervention. The study is structured as a two-arm, parallel-group randomized trial with a waiting list control. We propose recruiting a sample of 70 participants, randomly assigned to either an immediate intervention group, which will receive Emotion-Focused Therapy over twelve weekly sessions, or a control group that will receive the same therapy after a twelve-week waiting period. Data will be collected in the beginning, middle, and at the end of therapy, and in two planned follow-ups (three and six months after therapy). Once this protocol is implemented, if the therapy proves to be feasible, acceptable, and shows promising results, the findings will inform a large-scale randomized clinical trial to advance the understanding of this treatment for individuals aged 65 + .

## Introduction

Forgiveness has become a significant area of study in contemporary psychological research [1]. From a psychological perspective, we can distinguish between letting go—an emotional process through which the needs and feelings related to the offense are appropriately addressed without developing empathy towards the injurer—and forgiveness, which also involves the development of empathy or other more positive feelings, attitudes or perspective towards the injurer [2,3].

Although there is no consensual definition of forgiveness within the field of psychology [1,4], due to its multifaceted nature and diverse interpretations, we define it here as a process through which individuals address the negative emotions associated with an offense directed at them, while cultivating empathy and compassion toward themselves, the situation, and the offender [2,5]. It may encompass several stages: acknowledging the harm done, being open to the prospect of forgiveness, and nurturing the emotional, cognitive, and behavioral components essential for forgiveness [1,4].

Forgiveness is not the only path to emotional resolution; other approaches, such as letting go of resentment or validating emotions like anger, may also be effective [1,6,7]. In this context, resentment can be a response—among other possible reactions—to unjust and offensive situations. However, it can become detrimental and chronic if it persists and intensifies over time [1,2,8]. Following an offense, the absence of an adequate or adaptive response may leave the victim with unmet needs, such as the need for protection, support, and understanding [2]‌‌. Simultaneously, maladaptive responses may emerge, including silence, rumination, or aggression, which can develop into entrenched maladaptive patterns [2,8,9]. Such chronic resentment may lead to maladaptive behaviors, thoughts, and feelings, hindering a person’s ability to respond appropriately to the offensive experience [10,11].

Considering that forgiveness can be approached from the perspective of the victim, the offender, or through self-forgiveness, the present study focuses specifically on forgiveness from the perspective of the offended individual [4]. In light of the points discussed thus far, the study of forgiveness in older adults emerges as highly justified for several reasons. The ability to forgive develops and evolves over a person’s lifetime [12,13], even though only 5% of forgiveness research has included older adults in their samples [5]. This stage of life provides a valuable opportunity to address and resolve long-standing issues, particularly through processes of meaning reconstruction [6,14]. In fact, various scholars highlight that forgiveness can serve as a crucial resource throughout one’s life, helping to resolve interpersonal problems and contribute significantly to healthy aging [12,14]. Specifically, research has demonstrated that forgiveness offers a range of benefits during the aging process, with meaningful impacts across various dimensions of health [1,4].

At the physical level, forgiveness has been linked to a reduced risk of cardiovascular disease and to the enhancement of both endocrine and immune system functioning [1,14]. From an emotional perspective, it is associated with increased subjective well-being and lower levels of anxiety and depression [4,13]. Socially, forgiveness facilitates reconciliation with the past, fosters the development of empathy, and promotes stronger interpersonal relationships [1,13]. These benefits are frequently connected to reductions in stress, improvements in physical and psychological functioning, and enhanced social engagement—all of which are key components of successful aging [13,15]. Thus, forgiveness holds the potential to reduce the negative health effects of unresolved transgressions, playing a pivotal role in promoting well-being and resilience in older adulthood [14,13].

Forgiveness assumes a special relevance in later life, particularly in the context of resolving emotional injuries [6]. One contributing factor is the increased openness often observed with advancing age—manifested in a greater willingness to release resentments and to both extend and accept forgiveness. Scholars have noted that such tendencies are linked to indicators of positive aging and are inversely associated with signs of psychological and existential decline in late life [5,13]. At the same time, factors such as diminished social networks, growing physical frailty, the cumulative effects of individual life trajectories, and unresolved personal adversities may call for tailored consideration when addressing forgiveness processes in this demographic [4,6].

Indeed, several studies indicate that forgiveness has unique implications for older adults [13]. López et al. (2021) conducted a meta-analysis focusing on the effectiveness of forgiveness therapies among older adults, covering studies published from 1990 to 2020. Their analysis revealed that participants who underwent forgiveness therapies reported higher levels of forgiveness compared to those who did not [13]. Furthermore, these therapies were found to be negatively associated with levels of depression, stress, and anger, while being positively associated with indices of life satisfaction, subjective happiness, and psychological well-being [5,13].

Conversely, Webster et al. (2021) found that the severity of emotional injury resulting from transgressions is positively associated with a decline in the quality of physical health in older adults. Thus, forgiveness can diminish the adverse effects of transgressions on health, playing a significant role in successful aging [12,14]. Research by Toussaint et al. (2015) indicates an inverse correlation between forgiveness and markers of end-of-life health conditions, including cardiovascular, endocrine, and immune system functioning. Consequently, forgiveness has increasingly been recognized as a potential resource for promoting psychological well-being and longevity in later life, due to its positive associations with overall health and quality of life [12–14].

The growth of studies on forgiveness in older adults, along with increasing recognition of its positive impact, has paralleled the expansion of research on aging—driven by several factors, including the growing older population and advances in their quality of life [15]. Aging is a multifaceted process of personal development characterized by both gains and losses, influenced by various age-specific internal and external factors, individual traits, and the surrounding context [15,16]. This multifaceted process encompasses aspects such as fostering healthy lifestyles, acknowledging and adapting to inevitable losses, optimizing individual capabilities, and cultivating self-compassion [15].

Therefore, it is essential to understand aging as a multidimensional process involving the dynamic interaction of biological, psychological, and social components. Biological aging encompasses natural, progressive, and irreversible physiological changes [15]. Social aging reflects the roles, norms, and expectations shaped by cultural and societal contexts. Psychological aging includes not only potential cognitive decline but also gains in emotional regulation, accumulated knowledge, and self-awareness [15]. Aging is commonly categorized into three types: primary (normal), secondary (pathological), and tertiary (terminal) [16–18].

Contemporary gerontology increasingly adopts positive conceptual frameworks—such as successful, active, and healthy aging—which emphasize autonomy, social participation, and quality of life [15]. The World Health Organization (WHO) defines healthy aging as the process of developing and maintaining functional ability [18]. Psychological theories, ranging from Erikson’s stages of psychosocial development to more recent models like Gerotranscendence, underscore the developmental and transformative potential of aging [16,17]. Ultimately, aging well involves resilience, self-compassion, life satisfaction, social support, and living in accordance with one’s values [17].

Aging can involve psychosocial difficulties such as social isolation, loneliness, memory lapses, elder abuse, and dementia [17]. Moreover, the psychological well-being of this population is closely linked to autonomy, personal growth, self-acceptance, environmental control, health promotion, life goals, psychological care, and social relationships [15]. According to Erikson (1963), around age 65, individuals confront the final crisis: integrity versus despair. So, this period can be characterized by deep reflection, acceptance, and the integration of past events into a meaningful whole. Despair may manifest as an inability to reconcile life’s difficulties [18]. Given the above, we might hypothesize the significant role that forgiveness can play in fostering physical, mental, and emotional well-being as individuals age [1,4,15].

Although forgiveness may be associated with beneficial outcomes in adults over the age of 65, these results are not inherently assured. It is also important to consider the potential for pseudo-forgiveness and misunderstandings surrounding the concept of forgiveness [6,14]. Band-Winterstein et al. (2024), for instance, remind us that elder abuse—one of the primary psychosocial difficulties that tend to arise in aging [17]—involves a breach of fundamental trust and tends to be associated with specific feelings (anger, fear, sadness, revenge), thoughts (hostility, loss of respect for the aggressor), and behaviors (minimizing or engaging in aggressive actions, avoidance). Therefore, it is essential for individuals, in the process of forgiveness, to be aware and free to fulfill their fundamental psychological needs [4,6,14].

Concerning this, previous exploratory studies conducted by our research team [18,19] suggest that individuals over the age of 65 face specific challenges in the forgiveness process, including internal conflicts, rationalization, vulnerability, denial, and avoidance. These difficulties are often interrelated and interact in a dynamic way [18,19].

In light of these considerations—particularly the recognition of the harmful consequences of chronic resentment and the positive impact of therapeutic approaches in addressing such emotional injuries—there is substantial evidence supporting the benefits and positive outcomes of forgiveness-focused therapies [1,5]. Fundamentally, these interventions aim to enhance emotional well-being by addressing the emotional injuries resulting from interpersonal offenses. Specific interventions actively foster forgiveness, as exemplified by the REACH Forgiveness model [1] and the Enright Process Model of Psychological Forgiveness [4]. These two therapeutic models constitute the main pillars of forgiveness-related studies and interventions. The first model, which emphasizes the crucial distinction between the decision to forgive and the emotional process of forgiving, can be summarized in five key stages: recall the hurt (R), empathy (E), altruism (A), commitment (C), and holding onto forgiveness (H), under the acronym REACH [1]. The second model is characterized by its emphasis on the moral development of forgiveness-related reasoning and is structured into four main stages: uncovering, decision, work, and deepening [4]. Conversely, other approaches integrate forgiveness as one potential avenue for addressing emotional injuries. For instance, Emotion-Focused Therapy (EFT) presents the option of letting go—without necessarily involving forgiveness—as a viable strategy for healing emotional wounds [2,3].

A previous meta-analysis examining the effectiveness of psychotherapeutic interventions in promoting forgiveness [9] revealed that participants who underwent forgiveness-focused interventions experienced significantly higher levels of forgiveness compared to those who received no therapy and those undergoing alternative therapies. This meta-analysis conducted by Wade et al. (2014) highlighted not only the positive effects of forgiveness on well-being—such as reductions in anxiety and depression—but also demonstrated that the longer forgiveness-focused therapy is implemented, the greater are its benefits, with these positive effects tending to persist over time [9]. In addition, the analysis indicated that individually delivered interventions generally produce more favorable outcomes than group-based approaches [9]. Furthermore, the studies analyzed by Wade et al. (2014) indicated that EFT was one of the therapies with very positive results for resolving the emotional injury caused by an offense, as evidenced by the primary forgiveness measure (e.g., Enright Forgiveness Inventory–EFI) [10].

Therefore, it becomes clear that there is a need to develop new therapeutic approaches specifically tailored to adults aged 65 and older, thereby expanding the range of evidence-based interventions available for this population. In this context, EFT emerges as a promising approach, as suggested by Wade’s meta-analysis and its demonstrated effectiveness in other settings, and particularly due to its flexibility in addressing both *letting go* and forgiveness [9,11]. However, despite existing studies on EFT and forgiveness, this approach has not yet been tested with older adults [1,2].

Meneses and Greenberg (2019) propose an EFT protocol for the resolution of emotional injuries, which has been found to facilitate the forgiveness process by transforming painful feelings, thoughts, and actions into empathy, compassion, and love. The approach proposed by these authors involves five phases: establishing a therapeutic alliance; exploring emotions using chair work such as the empty-chair task; addressing self-interruptions through two-chair work; promoting forgiveness or letting go via emotional transformation; and concluding with strategies to reinforce emotion regulation and prevent relapse. The intervention was delivered over twelve weekly sessions [2]. According to EFT, therapeutic change occurs through activating painful emotions and memories and changing clients’ emotional experiences [3,7]. So, accessing disowned painful feelings related to the offense, including sadness, anger, and resentment, is crucial [5,8].

Now, EFT is highly effective for resolving internal conflicts by focusing on empathic attunement and emotions as central to therapy [2,3]. By accessing core primary emotions, it helps restructure painful experiences and their meanings, allowing for new emotional experiences to emerge [2,20]. This dynamic process adapts to individual needs, facilitating the resolution of unresolved conflicts [20,21].

Indeed, a variety of reasons—such as the need for an integrated understanding of forgiveness and aging, the scarcity of studies on forgiveness among older adults, the potential benefits of forgiveness, and the absence of EFT studies for older adults—justify exploring the impact of EFT applied to resolving emotional injuries in individuals over the age of 65.

Given all that has been mentioned so far, the importance of a study protocol in this context becomes clear, as it offers access to emerging research and guides the practical application of the findings [22,23]. Protocols can assist in outlining and justifying the research objectives, methods, participant criteria, interventions, and data collection, ensuring alignment with the study’s goals [24]. They support rigorous implementation, replicability, and efficiency while upholding scientific integrity, ethical standards, participant protection, and facilitating ethical approvals [25,26].

Thus, our current paper serves as preliminary research preceding a larger study, in which we aim to design a feasibility protocol for a RCT using EFT to resolve emotional injuries in individuals over 65 years of age.

### *Research objectives*‌‌

The present study is guided by the following objectives:

#### Primary objectives.

a. To assess the feasibility of the therapy and assessment protocol using key evaluation indicators, including recruitment rate, retention rate, and engagement rate.
b. To evaluate the acceptability of the therapy, considering the following indicators: treatment credibility and client outcome expectations, engagement, and satisfaction.

#### Secondary objective.

a. To explore the outcomes of EFT for the resolution of emotional injury among the study’s participants, according to forgiveness and letting go measures (primary outcomes) and depression and quality of life (secondary outcomes).

The aim of our study is to develop a methodologically sound and effective protocol that may serve as the foundation for a subsequent randomized clinical trial (RCT) to test the efficacy of EFT for the resolution of emotional injuries related to interpersonal offenses, in individuals aged 65 and older. In essence, a feasibility study such as the one proposed here, functions as a tool to assess the viability of the main trial (RCT)—specifically in terms of recruitment, retention, measurements, and other parameters—without necessarily focusing on clinical outcomes [22,27–29].

## Method

### Study design

The present publication is a Study Protocol, in the form of a feasibility study. This feasibility protocol is a two-arm, parallel, double-blinded, RCT aimed at informing a future trial assessing the effectiveness of EFT for resolving emotional injuries in older adults (aged above 65) in the context of interpersonal offenses.

This protocol has been registered on the ClinicalTrials.gov registry with number NCT06679452 (see section Ethics approval, below, for further details). The study plans to recruit 70 participants, with half randomly assigned to the EFT intervention and the other half placed on a waiting list to begin therapy after three months, during which they will be assessed [27]. Our chosen sample size aligns with previous similar studies [30,31]. Typically, for studies with two groups, the initial sample size should be adjusted to ensure an adequate number of completers (e.g., recruiting 40 to retain 30) [30,31]. Unlike randomized controlled trials, such feasibility studies do not require power calculations for effectiveness and instead focus on practical metrics, such as the ability to recruit a specified number of participants per month [30,31] (see more below). The recruitment process (which has not yet started) is planned for 2026, contingent upon securing funding. Following the two-month recruitment period, we anticipate initiating data collection. Subsequently, after a nine-month period, we foresee the presentation of results in a third phase. Data will be collected at the beginning, midway, and end of the therapy, as well as three and six months post-therapy (follow-ups). The study follows SPIRIT guidelines and CONSORT standards [28], consistent with prior research (Table 1) [22,29].

**Table 1:** SPIRIT checklist.

Section/item	Item N.º	Description	Addressed on page number
Administrative Information:			
Title	1	Descriptive title identifying the study design, population, interventions, and, if applicable, trial acronym	1
Trial registration	2a	Trial identifier and registry name. If not yet registered, name of intended registry	4
	2b	All items from the World Health Organization Trial Registration Data Set	N/A
Protocol version	3	Date and version identifier	2025
Funding	4	Sources and types of financial, material, and other support	N/A
Roles and responsibilities	5a	Names, affiliations, and roles of protocol contributors	1
	5b	Name and contact information for the trial sponsor	1 ccunha@umaia.pt
	5c	Role of study sponsor and funders, if any, in study design; collection, management, analysis, and interpretation of data; writing of the report; and the decision to submit the report for publication, including whether they will have ultimate authority over any of these activities	N/A
	5d	Composition, roles, and responsibilities of the coordinating centre, steering committee, endpoint adjudication committee, data management team, and other individuals or groups overseeing the trial, if applicable (see Item 21a for data monitoring committee)	N/A
Introduction			
Background and rationale	6a	Description of research question and justification for undertaking the trial, including summary of relevant studies (published and unpublished) examining benefits and harms for each intervention	5-6
	6b	Explanation for choice of comparators	5
Objectives	7	Specific objectives or hypotheses	5
Trial design	8	Description of trial design including type of trial (e.g., parallel group, crossover, factorial, single group), allocation ratio, and framework (e.g., superiority, equivalence, noninferiority, exploratory)	5-6
Methods: Participants, interventions, and outcomes			
Study setting	9	Description of study settings (e.g., community clinic, academic hospital) and list of countries where data will be collected. Reference to where list of study sites can be obtained.	6
Eligibility criteria	10	Inclusion and exclusion criteria for participants. If applicable, eligibility criteria for study centres and individuals who will perform the interventions (e.g., surgeons, psychotherapists)	9
Interventions	11a	Interventions for each group with sufficient detail to allow replication, including how and when they will be administered	11-15
	11b	Criteria for discontinuing or modifying allocated interventions for a given trial participant (e.g., drug dose change in terms of medication, participant request, or improving/worsening disease)	15-16
	11c	Strategies to improve adherence to intervention protocols, and any procedures for monitoring client adherence	15-17
	11d	Relevant concomitant care and interventions that are permitted or prohibited during the trial	N/A
Outcomes	12	Primary, secondary, and other outcomes, including the specific measurement variable, analysis metric (e.g., change from baseline, final value), method of aggregation (e.g., median, proportion), and time point for each outcome. Explanation of the clinical relevance of chosen efficacy and harm outcomes is strongly recommended	5, 15-16
Participant timeline	13	Time schedule of enrolment, interventions and assessments of participants. A schematic diagram is highly recommended (see Fig 2)	11
Sample size	14	Estimated number of participants needed to achieve study objectives and how it was determined, including clinical and statistical assumptions supporting any sample size calculations	6, 11
Recruitment	15	Strategies for achieving adequate participant enrolment to reach target sample size	15-17
**Methods: Assignment of interventions (for controlled trials)**			
Allocation:			
Sequence generation	16a	Method of generating the allocation sequence (e.g., computer-generated random numbers), and list of any factors for stratification. To reduce predictability of a random sequence, details of any planned restriction (e.g., blocking) should be provided in a separate document that is unavailable to those who enroll participants or assign interventions	11
Allocation concealment mechanism	16b	Mechanism of implementing the allocation sequence (e.g., sequentially numbered, opaque, sealed envelopes), describing any steps to conceal the sequence until interventions are assigned	11
Implementation	16c	Who will generate the allocation sequence, who will enrol participants, and who will assign participants to interventions	11
Blinding (masking)	17a	Who will be blinded after assignment to interventions (e.g., trial participants, care providers, outcome assessors, data analysts), and how	11
	17b	If blinded, circumstances under which unblinding is permissible, and procedure for revealing a participant’s allocated intervention during the trial	11
**Methods: Data collection, management, and analysis**			
Data collection methods	18a	Plans for assessment and collection of outcome, baseline, and other trial data, including any related processes to promote data quality (e.g., duplicate measurements, training of assessors) and a description of study instruments (e.g., questionnaires, laboratory tests) along with their reliability and validity, if known. Reference to where data collection forms can be found, if not in the protocol	10, 15-16
	18b	Plans to promote participant retention and complete follow-up, including list of any outcome data to be collected for participants who discontinue or deviate from intervention protocols	15-17
Data management	19	Plans for data entry, coding, security, and storage, including any related processes to promote data quality (e.g., double data entry; range checks for data values). Reference to where details of data management procedures can be found, if not in the protocol	16
Statistical methods	20a	Statistical methods for analysing primary and secondary outcomes. Reference to where other details of the statistical analysis plan can be found, if not in the protocol	17-18
	20b	Methods for any additional analyses (e.g., subgroup and adjusted analyses)	N/A
	20c	Definition of analysis population relating to protocol non-adherence (e.g., as randomised analysis), and any statistical methods to handle missing data (e.g., multiple imputation)	13-14, 18
**Methods: Monitoring**			
Data monitoring	21a	Composition of data monitoring committee (DMC); summary of its role and reporting structure; statement of whether it is independent from the sponsor and competing interests; and reference to where further details about its charter can be found, if not in the protocol. Alternatively, an explanation of why a DMC is not needed	16
	21b	Description of any interim analyses and stopping guidelines, including who will have access to these interim results and make the final decision to terminate the trial	18
Harms	22	Plans for collecting, assessing, reporting, and managing solicited and spontaneously reported adverse events and other unintended effects of trial interventions or trial conduct	17-18
Auditing	23	Frequency and procedures for auditing trial conduct, if any, and whether the process will be independent from investigators and the sponsor	17-18
**Ethics and dissemination**			
Research ethics approval	24	Plans for seeking research ethics committee/institutional review board (REC/IRB) approval	16, 18
Protocol amendments	25	Plans for communicating important protocol modifications (e.g., changes to eligibility criteria, outcomes, analyses) to relevant parties (e.g., investigators, REC/IRBs, trial participants, trial registries, journals, regulators)	18
Consent or assent	26a	Who will obtain informed consent or assent from potential trial participants or authorised surrogates, and how	18
	26b	Additional consent provisions for collection and use of participant data and biological specimens in ancillary studies, if applicable	N/A
Confidentiality	27	How personal information about potential and enrolled participants will be collected, shared, and maintained in order to protect confidentiality before, during, and after the trial	16,18
Declaration of interests	28	Financial and other competing interests for principal investigators for the overall trial and each study site	N/A
Access to data	29	Statement of who will have access to the final trial dataset, and disclosure of contractual agreements that limit such access for investigators	18
Ancillary and post-trial care	30	Provisions, if any, for ancillary and post-trial care, and for compensation to those who suffer harm from trial participation	N/A
Dissemination policy	31a	Plans for investigators and sponsor to communicate trial results to participants, healthcare professionals, the public, and other relevant groups (e.g., via publication, reporting in results databases, or other data sharing arrangements), including any publication restrictions	18
	31b	Authorship eligibility guidelines and any intended use of professional writers	N/A
	31c	Plans, if any, for granting public access to the full protocol, participant-level dataset, and statistical code	N/A
**Appendices**			
Informed consent materials	32	Model consent form and other related documentation given to participants and authorised surrogates	In the attachment
Biological specimens	33	Plans for collection, laboratory evaluation, and storage of biological specimens for genetic or molecular analysis in the current trial and for future use in ancillary studies, if applicable	N/A

### Study setting

The research will include individuals aged 65 years or older, of all genders, who meet the study’s inclusion criteria. Therapy sessions will be conducted individually and in person, in offices suited for psychological practice. Participants residing in senior housing will have the option, subject to agreement with their institution, to have sessions conducted in their facilities [22,23]. Each session will last one hour and occur weekly over a three-month period, resulting in a total of 12 sessions per participant, in accordance with the EFT protocol for resolving emotional injuries established by Meneses and Greenberg (2019), with adaptations presented below.

**Fig 1:**
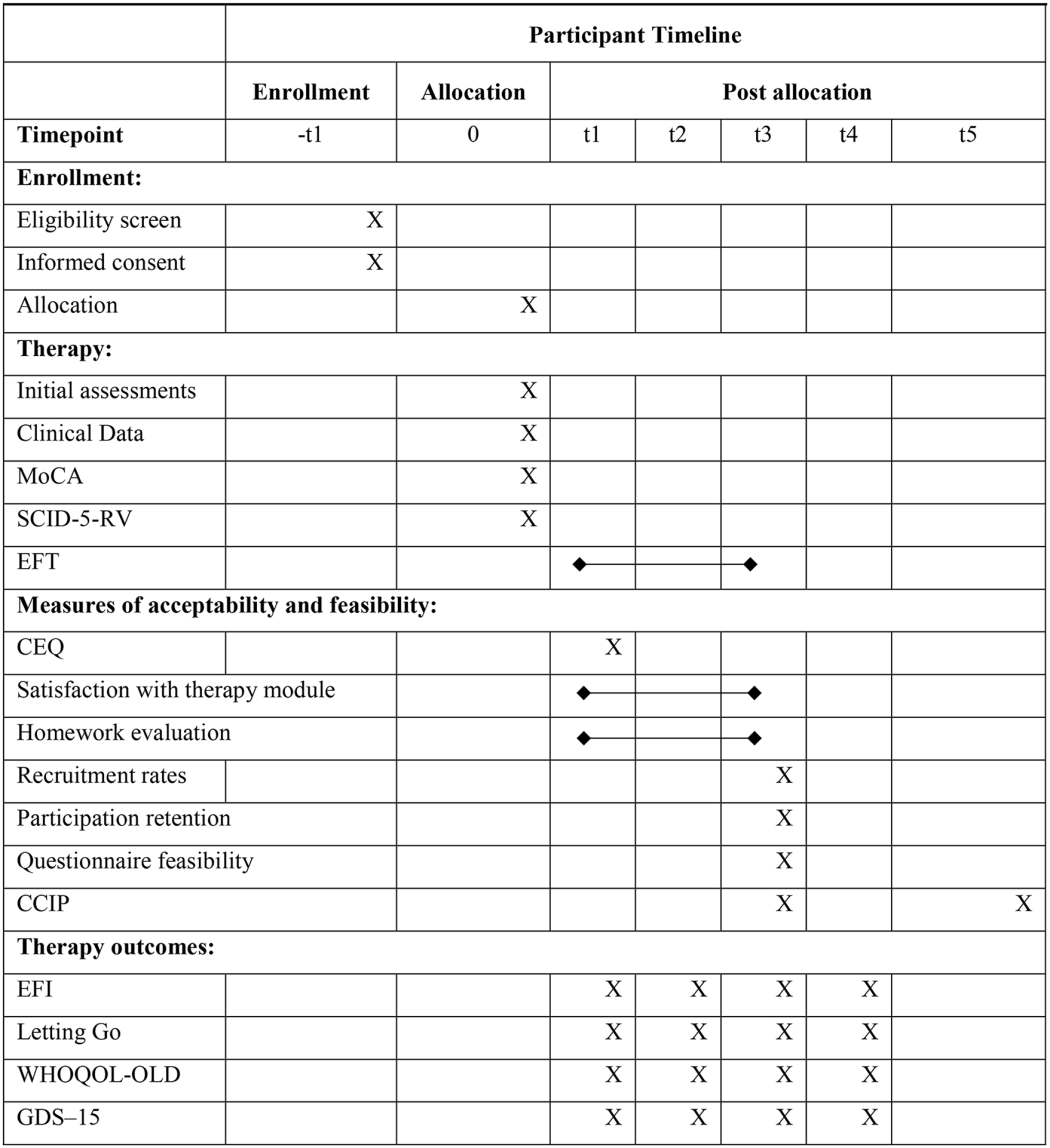
Study procedures.

At the conclusion of the designated sessions, in accordance with the study plan and study procedures (Fig 1), assessment data will be gathered in paper format. When a participant misses a session, it will be rescheduled as soon as possible. The questionnaires will be completed after the sessions. If necessary, a member of the research team will assist participants in completing them in order to prevent fatigue. In all cases, the questionnaires (with their number based on previous study protocols involving older adults [32]) will be administered in distinct and varied sessions, with clear instructions, scheduled breaks, and diverse formats to minimize fatigue — regardless of significant variations in the participants’ individual schedules [29,32]. All collected data—including attendance records and dropouts—will be analyzed in accordance with the study protocol (intent-to-treat analysis versus treatment completers analysis), not only to inform the current analysis but also to support the design of future studies. It is expected that at least 70 participants will complete the intervention (more on this below), and any reasons for dropout will be systematically documented. Regarding the secondary objective, which focuses on the analysis of outcome variables, particular attention will be given to the data from participants who complete the full intervention (completers).

If participants require extended support, provisions for longer therapy sessions may be made as a clinical decision [24–26]. For those who exceed the twelve-week period due to clinical reasons, the analysis will be split into two parts: one including all participants up to week 12, and a separate analysis assessing the impact of extra weeks for these participants only [22,23]. The current study protocol has been submitted to and approved by the Ethics Committee of the University of Maia, granting approval under the number 40/2022 (see Supporting Information: Ethics committee (in English and Original).

### Participants

Our feasibility study will include a minimum total sample of 70 older participants, with 35 randomly allocated to the intervention group (IG) and 35 to the waitlist control group (CG) [22,29]. Although feasibility studies do not require large samples [33], they should adequately represent the target population and be sufficiently large to fulfill the study’s objectives. Authors [33,30,31] agree that feasibility RCTs typically recruit between 24–50 participants per arm on average, with the exact number depending on the specific aims and objectives of the study. Larger sample sizes may be necessary for more complex or ambitious goals (in accordance with the G*Power requirements for comparing two groups) [33,30,31].

Given the exploratory nature of our study—aimed at evaluating the feasibility and acceptability of the therapy, as well as obtaining preliminary insights into its potential effectiveness among the participants involved, rather than establishing causality or generating generalizable results—we determined that a sample size of 70 participants would be appropriate. This decision was informed by precedents in comparable feasibility studies [22,29,34], as well as by practical considerations such as recruitment capacity, budgetary constraints, and the minimum sample size required to adequately address feasibility-related objectives [35]. Based on all of this, we propose to include 80 individuals in our study to ensure that at least 35 participants complete each comparison group (minimum N = 70). Therefore, in line with other studies, an additional 15 percent of participants will be recruited to account for typical dropout rates [9,13].

Therefore, considering the study’s objectives and similar methodological designs [22,29,34], our eligibility criteria include: being 65 years of age or older; having experienced an interpersonal offense; residing either at home or in a senior residence; stable use of prescribed psychotropic medication—defined as no changes in type, dosage, or frequency within the 8 weeks preceding study enrollment; a score of ≥ 26 on the Montreal Cognitive Assessment (MoCA); and fluency in either Portuguese or English. Exclusion criteria include: reporting an offense that occurred less than one year prior; the presence of severe emotional injuries (e.g., history of domestic, geriatric violence or abuse); psychiatric disorders (that would require a marked divergence in the treatment protocol), diagnosed according to DSM-5 criteria following a structured clinical interview (SCID-5-RV) conducted by a qualified professional; active suicidal ideation or parasuicidal behavior; the recent loss of a close family member (within the past two years); current participation in other therapeutic interventions or clinical studies; ongoing alcohol or drug abuse; cohabitation with another study participant; and frequent hospitalizations—defined as three or more admissions in the past 12 months, as documented in medical records [22,29,34].

### Recruitment

Client recruitment (not started yet) is planned to start in 2026, upon securing external funding. The recruitment, selection, and randomization process will be managed by an independent psychologist. Recruitment strategies include distributing posters and flyers in locations such as supermarkets, pharmacies, and senior living facilities, as well as through social media. The purpose of the recruitment posters and flyers is to inform and invite potential participants to engage in EFT, specifically targeting individuals over 65 years of age who have experienced interpersonal offenses. The central question posed will be: “Have you been the target of an injustice in the past that may still be affecting you emotionally?”

Those who express interest will be invited to a preliminary interview, conducted either in person or via phone/videoconference [29,36]. After obtaining a written consent, eligible individuals will be evaluated in a comprehensive assessment to confirm that they meet the study’s inclusion criteria. This includes administering the Structured Clinical Interview for DSM-5–Research Version (SCID-5-RV) to assess mental health conditions and MoCA to screen various cognitive domains [29,37,38].

### Randomization

**Fig 2:**
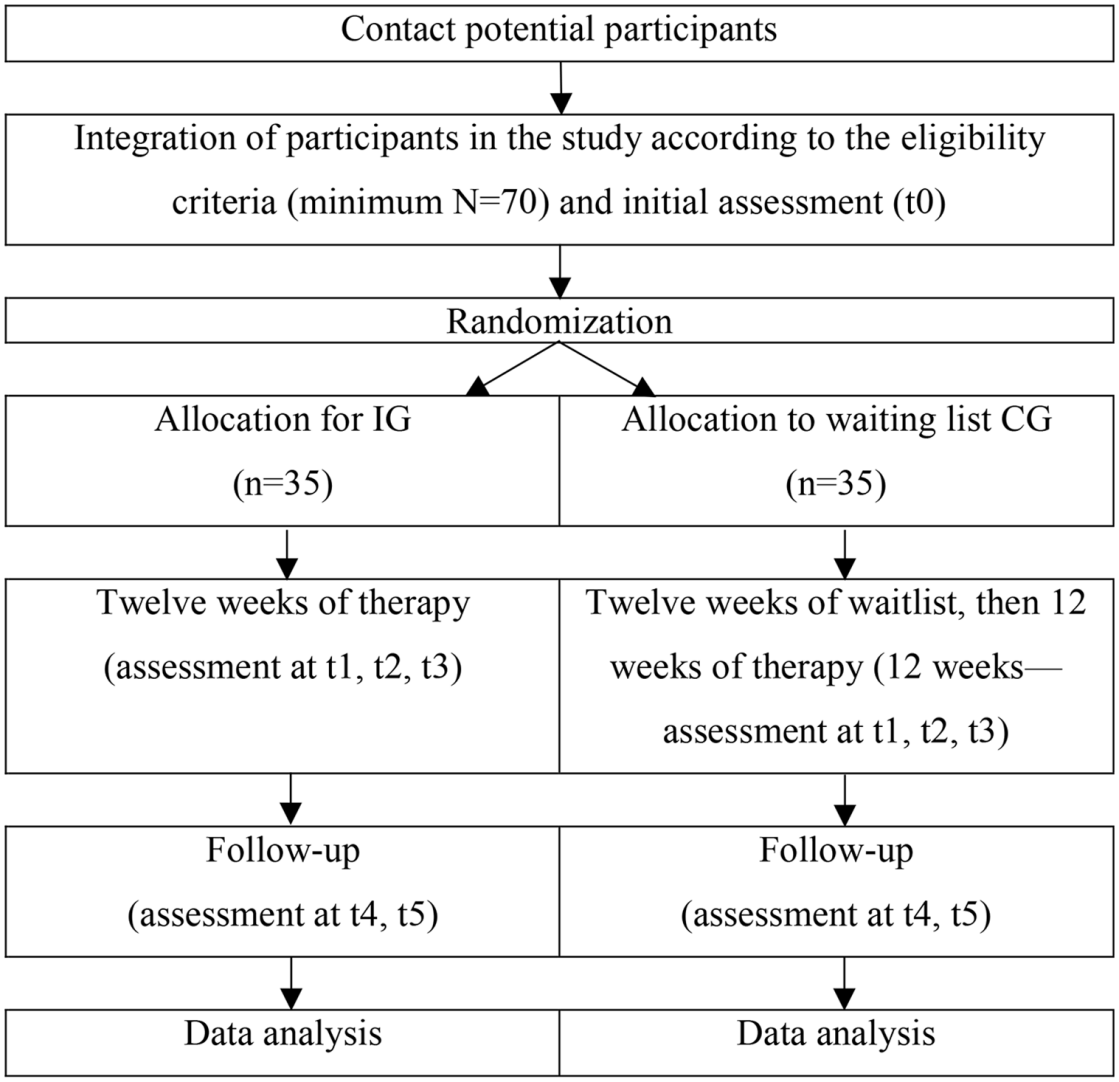
Overview of study processes.

Participants will be randomly assigned to either the immediate IG or the waitlist CG using a computer-generated block randomization method to minimize bias and ensure equitable allocation between groups [22,36,39]. Randomization will be enhanced through stratification based on key participant characteristics—age, gender, educational level, and spirituality—to ensure a balanced distribution across both groups [9,29]. The CG will not receive any information about the therapy or recommendations to preserve the integrity of the CG. The allocation process will be double blinded, with group assignments concealed from both the principal investigator and the participants (Fig 2) [27,39].

### Therapy

Participants will be offered twelve in-person sessions, scheduled once a week. The therapy is structured into five phases (Table 2), following the protocol proposed by Meneses and Greenberg (2019). This approach emphasizes emotions in the therapeutic process and addresses the primary difficulties reported by adults in relation to forgiveness. Each session will be adapted to meet the specific needs of the clients.

**Table 2:** EFT for the resolution of emotional injuries session: An overview.

Phase	Content	Home Practice
Phase I:	Creating an alliance	Diary of feelings towards the injurer.
Phase II:	Evocation and exploration	Particular attention can be given to awareness of emotional experiences
Phase III:	Self-interruption work Empty-chair work	Drafting the letter to the injurer and its response (for personal use only); encouraging reflection and the identification of difficulties in forgiveness
Phase IV:	Empowerment and letting go or forgiving	Narratives of new meanings of the experience and the injurer
Phase V:	Termination	

#### Phase I: Creating an alliance.

The initial phase of therapy emphasizes the establishment and reinforcement of the therapeutic alliance to cultivate a safe, exploratory, and empathic environment. This phase is used to validate the emotional injury reported during the initial assessment and to clarify its most challenging aspects, through an empathic attunement to painful aspects of the interpersonal offense and painful emotions [2]. The therapeutic process dynamics are presented to the client, emphasizing the focus on difficulties arising from the experienced offense. Once the client feels ready, the therapeutic relationship is sufficiently solid, and the marker of an unresolved, emotional injury related to a past interpersonal situation is identified, the empty chair task can be introduced [19,40]. This task involves the client visualizing the injurer in an empty chair, to facilitate the exploration, awareness, expression, and processing of painful, core primary emotions to address unmet needs and resolve emotional injuries [20,40]. It is a collaborative process between the therapist and the client, with a particular emphasis on transforming the primary emotions associated with the emotional injury [2,7].

#### Phase II: Evocation and exploration.

The second phase of therapy primarily involves accessing and addressing problematic emotional processes and the needs that may arise from them. Therapists try to identify emerging markers, namely, feelings of pain, sadness, anger, vulnerability that clients may feel when remembering what happened or what offended them [2,20]. Along with the empathic and exploratory stance of the therapeutic process, the empty chair task continues to be proposed when markers of unfinished business appear, to help participants deal with the lingering feelings of pain due to the emotional injury. The task facilitates the arousal and exploration of emotions, recognition and differentiation of feelings, and the identification of various forms of anger. It can also assist clients in identifying secondary or non-adaptive emotions while accessing primary emotions [20]. Thus, the empty chair task can facilitate clients’ connection with and expression of their unmet emotional needs [2,8].

#### Phase III: Self-Interruption work.

The interruption of experienced emotions is a prevalent challenge in addressing unfinished business through the empty-chair task [2,40]. Consequently, there is often a necessity to address self-interruptions during the deepening phase of the empty chair task. So, this third phase specifically aims to assist participants in recognizing markers of self-interruption, typically manifested as emotional constriction, resignation, or hopelessness [2]. To deal with self-interruptions, we employ the therapeutic task of two-chair enactment, which entails engaging in an imaginative dialogue between different parts of the self: the part that interrupts or obstructs and the experiencing self, who endures the consequences of this interruption [40]. Through this approach, clients can gain awareness and access how they self-interrupt, and through which processes self-interruptions occur, often rooted in experiential avoidance of more painful emotions. This enables to address, among other emotional processes, feelings of hopelessness and discouragement that may emerge from the self-interruption process [2].

#### Phase IV: Empowerment and letting go or forgiving.

This stage of the process aims to deepen emotional processing through the empty-chair task, by accessing painful, chronic (primary maladaptive) emotions, and their underlying unmet needs [20,41]. This emotional deepening process can facilitate the recognition and expression of the core pain [2,41]. Therapists aim to facilitate clients’ mourning of unmet needs, promoting opportunities to fulfill those needs, and fostering new emotional experiences (such as compassion and pride in oneself) and new perspectives on the offense and the injurer [2]. The forgiveness process usually involves experiencing new emotions (e.g., empathy towards the injurer) and changing the view of the injurer, that becomes softened, for example, through an understanding of the reasons underlying the offense. The emotional transformation of resentment can happen through an emotional response that involves compassion and love [2]. The process of resolving emotional injuries can also be achieved through *letting go*, which is understood as a process involving the addressing of unresolved emotional needs and feelings related to the offense and the offender. *Letting go* entails acknowledging and working through the emotional impact—such as pain, resentment, or anger—without necessitating the development of empathy or positive feelings toward the offender. In contrast to forgiveness, which typically involves a relational shift toward the offender, *letting go* focuses on internal emotional healing, aiming to alleviate distress while maintaining personal psychological boundaries [2].

#### Phase V: Termination.

The therapy seeks to address several key dimensions: the client’s role as an agent of their own emotions; the experience of change as a continuous process; the acceptance of separation and loss; and the prevention of potential relapses [2]. Ultimately, the therapeutic process aims to culminate in forgiveness, which involves an adjusted regulation of the expression of resentment, anger, and shame associated with the offense, alongside the promotion of compassion. Alternatively, the therapy may conclude with a process of letting go, which entails a thoughtful processing of unmet needs and feelings related to the offense and the offender, without necessarily fostering empathy or compassion toward the latter [2].

### Anticipated adjustments of this protocol for older populations

Considering the current literature [6,14], including our own studies [18,19], we estimate that the older population may face specific difficulties in the process of forgiveness and resolution of emotional injuries. So, we anticipate a set of specificities and adaptations to the Meneses and Greenberg (2019) protocol.

#### Phase I specificities.

Studies indicate that psychosocial difficulties, such as social isolation or elder abuse [17], can lead individuals to minimize interpersonal offenses and their respective emotional injuries [6]. For example, a person who is dependent upon a family member may not express their true feelings and may (superficially) forgive to avoid negative interpersonal consequences. While recognizing the importance of valuing current relationships, therapists may need to facilitate clients’ awareness of the significance of acknowledging their own suffering and the negative consequences of emotional injury, as blocking the emotional process can lead to additional problems (much like the contrast between normative versus complicated grief, as proposed by Sharbanee & Greenberg, 2023).

To address this issue, we propose that therapists undertake experiential formulation and focusing as a therapeutic strategy aimed at highlighting the importance and value of accessing avoided emotions, alongside the promotion of greater self-awareness and self-acceptance throughout the therapeutic process and beyond [20,41]. This aims to explore client difficulties in experiential terms, emphasizing therapy’s focus on personal feelings and not necessarily on changes on relationships (i.e., forgiveness does not imply reconciliation) [22]. Indeed, the intention is to allow individuals to access their experiences more freely and authentically, without necessitating behavioral changes or causing negative repercussions [18,19,41].

#### Phase II specificities.

Previous studies by the present authors [18,19] have identified specific difficulties (or markers) that older individuals may face in the process of forgiving. One such difficulty is avoiding painful experiences by being purely conceptual. For instance, an older person might say, “I feel neither anger nor sadness [...] Forgiving is like lifting a weight off my shoulders [...] I don’t feel the need for support or affection.” This type of narrative is often characterized by an overly intellectualized self-voice that suppresses a more vulnerable, experiential voice, which may signal the presence of self-interruption [18,20,41]. In therapy, tasks such as empathic exploration [40] and focusing [42] can be proposed to encourage grounding in the experience. Subsequently, the two-chair enactment task [43,44] can be employed to facilitate dialogue between the dominant voice and the disowned voice.

#### Phase III specificities.

A previous study with older people [19] showed that all participants struggled with internal conflicts in the process of forgiveness. An example narrative is: “To me, forgiving means forgetting and never dwelling on it again [...] when I forgive, I don’t forget, because the memory resurfaces, along with the hurt and sorrow.” These conflicts are characterized by a persistent divergence between two dimensions (or voices) of the self that do not communicate with each other [18]. This marker—self-conflict or negative self-treatment—can be addressed in therapy through the two-chair dialogue task, aiming for a satisfactory negotiation or understanding between the conflicting parts [7,43,45].

Another difficulty we anticipate is avoidance by suppression of the painful experience [18]. Consider the following example: “If I am shocked, it passes. I don’t think, I forget, I focus on other things. There is so much to live for [...] I can keep control.” This may reflect an automatic tendency to deny or avoid the emotional injury caused by the offense, hindering recognition of the interruption process and access to the repressed internal experience. We identify this marker as a potential process of self-interruption, often involving immersion in painful memories and a focus on other dimensions or problems [18,19]. The two-chair enactment task can be beneficial in bringing the automatically interrupted voice into action [43,44].

Another challenge we foresee is that older individuals might delegate the responsibility of forgiveness to others, including a divine entity [18]. For example: “Thinking about God helps, because it gives me peace, relieves me [...] The one who forgives is God.” In line with what has been observed in our studies [18,19], this response may indicate a collapse of the self in reaction to profound despair caused by the emotional injury [20,46]. This type of despair can be characterized by a sense of loss of agency, where one feels entirely powerless and submits to another (e.g., delegating forgiveness, justice or repair to a divine entity), demonstrating profound demobilization [18]. Considering this marker, tasks such as empathic affirmation [41] and compassionate self-soothing [47] can be beneficial.

#### Phase IV and Phase V specificities.

Based on our prior research [18,19], it is important to emphasize that certain emotional experiences resulting from therapy can indicate if the therapeutic process is progressing positively. These encompass physiological indicators like serenity, tranquility, and calmness; cognitive shifts such as reduced rumination, enhanced learning, and decreased negative thoughts; emotional transformation including peace, joy, and relief; behavioral adaptations towards oneself, others, and the environment; and self-transformation, characterized by satisfaction, the cultivation of new internal narratives, heightened empathy and compassion, and receptiveness to transcendence [5,13,18,19].

We anticipate that transforming the emotional difficulties associated with emotional injury and fostering self-narrative reconstruction may pose a significant challenge for this population. This age group is particularly susceptible to promoting satisfactory personal integration and enhancing positive life meaning, which are crucial developmental tasks at this stage of life [48]. Indeed, we see this as a consolidation of change, a reconciliation of the self with one’s life, necessitating specific narrative-meaning reconstruction work, as outlined in the self-narrative reconstruction task [19,20], which we consider particularly suitable for this population [6,13].

### Therapists

Sessions will be administered by psychologists with several years of psychotherapy training research and clinical practice in EFT. The first author–Bernardo Corrêa d’Almeida–holds a PhD in Theology and a PhD in Psychology (specialization in Clinical Psychology), and is a psychologist with previous training in EFT [18,19,49]. The second author–Carla Cunha–is a psychologist with a PhD in Psychology (specialization in Clinical Psychology). She is a specialist in Clinical and Health Psychology, holding an advanced specialization in psychotherapy, as recognized by the National Psychologists’ Association. Furthermore, she is a certified EFT therapist and supervisor, as accredited by the International Society of Emotion-Focused Therapy (isEFT). She provides supervision and training in EFT and will serve as one of the ongoing supervisors for the team. Additionally, she has authored several scientific articles in the field [18–20].

Our therapy team will also involve two more psychologists with training in EFT, involved in the PhD program of the university. All therapists will be trained in this protocol [2] and supervised by a certified EFT supervisor. The therapists, with a commitment to avoiding any form of ageism, will be encouraged—through peer supervision—to address the key vulnerabilities, needs, and potential of this client population age group [6,15]. In addition to the four psychologists who are part of the therapy team, this research will involve another psychologist who will be responsible for the recruitment process and the assessment procedures. This psychologist—to safeguard the reliability of the evaluation results—will not be involved in the therapeutic process.

### Therapy fidelity

The team of therapists will be provided with and instructed on a therapy protocol. They will also receive a checklist outlining specific procedures typical of each therapy phase. Therapy sessions will be audio and video recorded, and a selection of these recordings will be randomly analyzed by psychologists with supervisory experience in EFT to ensure adherence to the model and fidelity of therapy procedures. Therapists will receive supervision from a qualified EFT supervisor, which will involve corrective feedback to ensure therapist adherence and competence in relation to the EFT model [20,41]. To evaluate how closely therapists follow the EFT model, we will use the Person-Centered and Experiential Psychotherapy Scale (PCEPS-EFT-9) [50]. This scale evaluates treatment integrity in EFT, namely a qualitative assessment of the adherence to protocol and competence level for each EFT therapist, based on their observable performance (using a Likert scale from one to six in nine distinct competencies).

### Participant retention

To minimize possible abandonment of therapy, those involved in the study will always be alerted in advance about their appointments and will have the possibility of rescheduling them if clients are unable to attend [22,29].

### Assessments and measures

Several questionnaires will be used to assess the acceptability, feasibility, and to explore the outcomes of the therapy—see Figs 1 and 2.

#### Intervention acceptability measures.

*Therapy Credibility and Expectancy*

We will use the Credibility/Expectancy Questionnaire (CEQ) [51,52] to measure treatment credibility and client outcome expectations. The CEQ is a self-report questionnaire composed of six items grouped into two subscales: treatment credibility and expected outcome–on a 9 item Likert scale. Credibility assesses the degree of confidence that participants have in the usefulness of therapy as treatment for their difficulties. Client outcome expectations assesses how much participants believe they can benefit from the therapy protocol that is provided [52].

*Session Satisfaction Module*

To evaluate participants’ assessment of each specific session of the intervention protocol, they will be asked at the end of each session to rate how beneficial they found each session and corresponding homework activity. This will be done through a simple question, with responses varying on a 5-point Likert scale [22].

*Global Therapy Satisfaction*

To assess participants’ satisfaction with the global intervention, we will use the Client Change Interview Protocol (CCIP) [40,53]. This semi-structured interview, comprising open-ended questions, has a duration of approximately 60 minutes and is administered at the conclusion of therapy (at t3 and t5). The CCIP explores potential processes of change experienced by clients during therapy, covering attributions, meanings, and relevance. Participants evaluate specific changes they have experienced using a 5-point scale, assessing the extent to which these changes were expected, likely without therapy, and their perceived value [54]. Any negative evaluations derived from the Session Satisfaction Module will also be examined. Consequently, these procedures are intended to facilitate necessary adjustments to the treatment protocol, thereby enhancing its feasibility and acceptability based upon client feedback.

#### Intervention feasibility measures.

*Recruitment rates*

Refers to the number of participants able to join the therapy as a result of the recruitment process.

*Participation rates*

Participants who completely discontinue therapy should be classified as dropouts.

*Questionnaire feasibility*

The feasibility of the questionnaires will be measured by evaluating the completion rates of the questionnaires including client feedback and the degree of missing data.

*Homework evaluation*

To assess the value of homework, an adapted version of the Homework Rating Scale (HRS) [55] will be employed, consisting of seven questions rated on a 5-point Likert scale. Participants will be asked to evaluate their level of achievement, understanding, effort, utility, relevance, satisfaction, and the impact of the exploratory tasks assigned during the therapy weeks [22].

#### Exploratory outcomes.

The exploratory results encompass the primary outcome variables of forgiveness and letting go, as well as the secondary outcome variables of quality of life and depression.

*Enright Forgiveness Inventory*

The Enright Forgiveness Inventory (EFI) [10] is a self-report measure designed to assess the level of forgiveness in response to a specific offending situation. The scale begins by asking participants to visualize the personal offense and imagine what happened. In a second phase, participants are asked to describe the offense in their own words. The EFI scale comprises 60 items organized into three 20-item subscales that measure affect, behavior, and cognition related to forgiveness, and a final 6-point Likert scale known as the Attitude Scale. Finally, the EFI concludes with a final question assessed on a 5-point Likert scale, which evaluates the extent to which clients report having forgiven their injurer.

*Letting Go Measure*

The Letting Go Measure [3] is a self-report assessment comprising a single item. It evaluates the extent to which individuals have let go of their negative emotions towards the injurer. Scores range on a Likert scale from 1 to 5 points.

*Quality of Life*

The European Portuguese World Health Organization Quality of Life Assessment in Older Adults (WHOQOL—OLD) [55,56] comprises 28 items classified on a five-point scale and encompassing seven domains: sensory, autonomy, activities, social, death, intimacy, and family life [56].

*Geriatric Depression Scale*–*15*

The Geriatric Depression Scale–15 (GDS-15) [57,58] is specifically designed for screening depressive symptoms in older adults. It consists of 15 straightforward questions, each requiring a simple Yes or No response. The scale ranges from zero (indicating no depressive symptoms) to 15 points (representing the highest severity of depressive symptoms), with each affirmative response scored as 1 point. The cut-off points are defined as follows: 0–4 for the absence of depressive symptoms, 5–8 for mild symptoms, 9–11 for moderate symptoms, and 12–15 for severe symptoms [58].

### Data handling and storage

The data protection plan, approved by the Ethics Committee of the University of Maia—with the ethical board granting approval under the number 40/2022—ensures that participants are informed about the study’s objectives, confidentiality, and data management procedures, and sign an informed consent form. Consent forms will be securely stored in the corresponding author’s office. Participants will be assigned codes, and their names kept separate from other data. The collected data will be stored in a locked box within a securely closed cabinet, to which only the author has access. This cabinet is located in the investigator’s personal workspace, with no access permitted to third parties. Data will be digitized for analysis, with stringent accuracy checks and password-protected access for trial staff. Audio and video recordings will be securely stored and destroyed six months post-therapy. Any changes to data handling will be reviewed by the ethics committee [22,29].

### Data collection

As illustrated (Fig 2), various measures will be administered throughout the study—from participant selection to the two planned follow-up assessments. To minimize the burden on participants, questionnaires scheduled for completion at the end of the therapy will be distributed either during the final session or at home between sessions. The questionnaires will be returned in sealed envelopes to the therapists, who will then hand them over to the psychologist responsible for the evaluation process. This independent psychologist will also conduct the initial assessment for study integration and the final qualitative interview [22,29].

To efficiently assess whether potential participants meet the study criteria, eligible individuals will undergo the Structured Clinical Interview for DSM-5—Research Version (SCID-5-RV) [59] and the Montreal Cognitive Assessment (MoCA) [37,38]. Additionally, socio-demographic data, including age, gender, occupation, religious beliefs, current medications, and details regarding the interpersonal offense or emotional injury experienced, will be collected at the outset [22,25,34].

After participants are assigned to their respective groups, three assessment points will be established: before therapy begins, at the sixth session, and at the final session. These assessments will focus on Forgiveness, Letting Go, Quality of Life, and Depression. Before therapy commences, the CEQ will also be administered. Following each session, the Satisfaction Therapy Module and Homework Evaluation will be conducted. Upon completion of therapy (t3 and t5), the CCIP will be administered. To ensure adherence to the therapy protocol, sessions will be recorded for subsequent supervisory evaluation [22,29,34]. Research assistants will provide updates and reminders for follow-ups via telephone and/or email.

### Statistics and data analysis

#### Quantitative analysis.

Quantitative data analysis will be conducted using SPSS version 29.0 for Windows [60]. Given the study’s exploratory character, particularly with regard to its secondary objective—namely, the study of treatment outcomes—the primary focus is not to test formal hypotheses but rather to provide an initial descriptive overview of the data [22,25]. Accordingly, we have chosen to employ descriptive statistics such as means, standard deviations, frequencies, and percentages to summarize the results. By prioritizing descriptive statistics, we seek to maintain methodological rigor while offering meaningful insights that may inform future, more comprehensive and definitive investigations [35,34].

The ratio of individuals who accepted the invitation to participate in the study relative to the total number of formal invitations sent will help determine the recruitment size. It is anticipated that accurately quantifying the total number of individuals reached through various channels may prove challenging. Additionally, the ratio of participants who complete therapy compared to those who initially commenced it will offer insights into retention rates. Furthermore, the completion rates of the questionnaires will serve as an indicator of their feasibility. Other measures, including assessments of acceptability and feasibility—such as the CEQ, CCIP, Session Satisfaction Module, and Homework Evaluation—will be assessed through exploratory data analysis [22,25,29,34].

To identify and quantify potential associations among the different variables for the main trial, Pearson correlations will be performed to analyze observed changes over time in the control group for all outcome variables. The calculation of the sample size for a future large-scale randomized controlled trial will be guided by the statistical power required to detect clinically meaningful effects, rather than by the provisional estimates derived from this feasibility study [35].

#### Qualitative analysis.

The data collected from the CCIP will be recorded. After being transcribed, it will be subject to respective analysis.

### Progression criteria

This study protocol will be developed for broader research based on the assessment of its acceptability and feasibility. Regarding the CEQ measure, the study will proceed without changes if the result is ≥ 6 on a 9-point Likert scale. The study will also progress without changes in the Session Satisfaction Module if the result is ≥ 3 on a 5-point Likert scale [22]. For recruitment, the progression criterion will be a rate of 75–100% of the sample size [61]. The criterion for retention level will be a rate >80% [36]. The progression criterion for the questionnaires will be a response rate >90%. For homework assessment, a score of ≥ 3 on a 5-point Likert scale will be required [22,29].

### Ethics approval and consent to participate

The protocol is registered with number NCT06679452 on the ClinicalTrials.gov registry. It adheres to the ethical standards outlined in the Code of Ethics of the Portuguese Association of Psychologists (aligned with the American Psychological Association). This protocol was submitted to and received approval from the Ethics Committee of the University of Maia (under the reference number 40/2022). Participants will be fully informed about the study’s context, goals, and methodologies before giving written informed consent to participate. Participation will be voluntary, and participants can withdraw at any time and request a summary of the findings, upon their participation.

### Dissemination

The study results will be published in high-impact journals and presented at international conferences. Participants may receive the results if interested, provided in a customized document. This study is expected to lay the groundwork for a larger protocol, ultimately leading to a randomized clinical trial with appropriate partnerships and funding.

## Discussion

The increasing older population and associated challenges underscore the importance of prioritizing their emotional well-being. EFT could be essential in personal primary care for the older population, as well as within their families and communities [14,13]. Given the significance of forgiveness for the quality of life of older adults, and the lack of EFT studies focusing on forgiveness and the resolution of emotional injuries in this demographic, this feasibility study aims to advance psychological research in these areas.

Feasibility studies, as initial research steps, estimate key parameters before main studies [29].They outline optimal conduct for RCTs, lacking the statistical power to justify effects [62]. Many authors emphasize the importance of feasibility studies for future RCTs, including assessing acceptability and feedback of participants [22], which is contemplated here through several measures (self-report and interviews). Older individuals are underrepresented in clinical trials, with only 7% of RCTs in 2012 specifically studying older adults [63], which reinforces the potential contributions of this study to the literature.

Despite the potential of this study, it has limitations. The sample size (N = 70) restricts generalization and conclusions regarding the effectiveness of the therapy, necessitating caution in interpretation. Longer follow-up periods could enhance the study by providing more robust data on the impact of the therapy; however, implementing such follow-ups may be challenging within this population. Additionally, ensuring uniform characteristics among sample members and across groups may prove difficult [22]. In summary, aging and forgiveness are increasingly significant in people’s lives. Demonstrating the feasibility and acceptability of EFT and forgiveness could significantly promote the quality of life for older individuals and their contexts.
